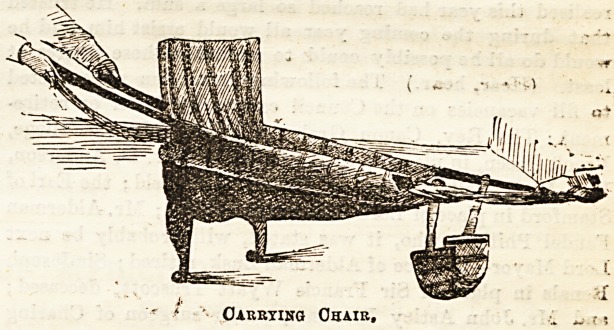# Practical Departments

**Published:** 1895-11-30

**Authors:** 


### PRACTICAL DEPARTMENTS.

### A NEW LIFTING SEAT AND CARRYING CHAIR.

The want of a simple and yet absolutely strong carrying chair is often felt by those in charge of more or less helpless invalids, the price of many of the more elaborate kinds being often prohibitive, and their own considerable weight adding to rather than diminishing the inevitable difficulties of conveying, perhaps, a heavy patient up and down stairs, or from bed to sofa and *vice versâ*. Ideas similar to that carried out in the chair of which we give an illustration have occurred to practical people and been made use of in private houses before now, but nothing so complete in every detail has been actually brought into the market for general use until quite recently, and for it the public are indebted to a lady who has had herself experience in sickness and nursing, and to whose practical common-sense and ingenuity this most useful invention is due.

It is really necessary to see the thing itself to appreciate the intricate simplicity, if one may so put it, of the loops and hooks which make for the comfort and security of its occupant, and ease of movement to the carriers; but the printed particulars are so clear that it will be well to give them here at length. The combined and folding lifting seat and carrying chair is made in canvas or carpet, and consists of three parts, having two sets of poles (one set shorter than the other, for simple lifting from one chair or sofa to another). When used as a carrying chair the back and adjustable foot-rest hook on and off as required. The back hooks to the seat in five places—three at the back of the seat and two at the sides—which fasten to the small loops through which the holders run. The foot-rest hooks on to the corresponding loops through which the other holders run. The long poles, 48 inches long, are then passed through the loops of the foot-rest, the hems of the seat, and the loops of the sides at the back. The holders are only needed when the longer poles are used to keep the seat in place; and they, like the back and foot-rest, hook on and off as required. If it is wished to raise the front of the seat (as is desirable in carrying anyone up and down stairs) the poles are passed not only through the hems of the seat, but also through the loops under the front of it; this shortens and raises the front. Long loops of strong braid are attached firmly to the sides, so that the bearers at either end may have an extra safeguard towards keeping the patient in position. The back and foot-rest are, of course, not needed when the seat is used with the short poles merely for a lift.

Just now a specimen chair is on view at the Ladies' Sanitary Association, 22, Berners Street, where Miss Adams will

Carrying Chair.

explain all particulars. The chairs may be had in canvas or carpet, the whole affair complete in canvas from 8s. 11d., and in carpet from 13s. 6d.; and they may be obtained from Messrs. Fish and Son, Suffolk House, Ipswich; or Messrs. Bullin and Son, 86, High Street, Tunbridge Wells. It is hoped soon to have them on sale at the Civil Service Stores, Haymarket, and at other places in London and elsewhere, and the demand for them ought to be considerable when once they are made known. Judged on its merits the contrivance is an excellent one.

A correspondent suggests that such a carrying chair as we have described might be usefully employed in the Ashantee Expedition, in conjunction with the medical service.

## Figures and Tables

**Figure f1:**